# Supplementary material for: Social contact as a strategy to reduce stigma in low- and middle-income countries: A systematic review and expert perspectives
Source: PLOS Glob Public Health. 2024 Mar 27;4(3):e0003053. doi: 10.1371/journal.pgph.0003053 (PMC10971769; doi:10.1371/journal.pgph.0003053)
Supplement: S1 Text — (DOCX) [file pgph.0003053.s003.docx]

**Supplementary material S1 Text: complete search strategy and inclusion/exclusion criteria**

*Search strategy*

- The following databases are searched: Academic Search Premier, Anthropology Plus, CINAHL, Cochrane Library, Embase, ERIC, PsycInfo, PubMed, Scopus, SocINDEX, Sociological Abstracts, and Web of Science
- The full search strategy is as follows: stigma **AND** social contact **AND** intervention **AND** setting*.* All used search terms can be seen below.
- MeSH terms are only used in the medical databases where allowed: PubMed, Cochrane Library. For the other databases, the search strategy (as provided below) is entered without the MeSH terms.
- Filters were applied when applicable:
  - Languages: English, Dutch, German, French, Spanish
  - Human participants (not animals)
  - Publication type: primary research and peer-reviewed

| **Database** | **Search date** | **Stigma searched in** | **Social contact searched in** | **Intervention searched in** | **Setting searched in** | **Filters applied** | **MeSH terms** |
| --- | --- | --- | --- | --- | --- | --- | --- |
| **Cochrane Library** | 18-02-2022 | Title | Title/abstract/ key words | Title/abstract/ key words | Title/abstract/ key words | n/a | Yes |
| **EBSCO (Academic Search Premier, Anthropology Plus, CINHAL, SocIndex)** | 17-02-2022 | Title | Abstract | Abstract | Abstract | Yes: peer-reviewed | No |
| **OVID (Embase, ERIC, PsycInfo)** | 18-02-2022 | Title | Title/abstract | Title/abstract | Title/abstract | Yes: humans | No |
| **PubMed** | 18-02-2022 | Title | Title/abstract | Title/abstract | Title/abstract | Yes: humans, languages | Yes |
| **Scopus** | 17-02-2022 | Title | Title/abstract/ key words | Title/abstract/ key words | Somehow n/a, therefore Scopus searched without *setting* | Yes: publication type (article, review), language | No |
| **Sociological abstracts** | 18-02-2022 | Title | Abstract | Abstract | Abstract | Yes: language, peer-reviewed  Excluded: document types (dissertations/thesis, book, conference paper) | No |
| **Web of Science** | 18-02-2022 | Title | Abstract | Abstract | Abstract | Yes: document types (article, review, early access), language | No |

***Stigma*** *search terms*

stigma* OR prejudic* OR discriminat* OR stereotyp* OR ostraci* OR expel* OR out-group* OR "out group" OR outgroup OR "social exclusion" OR "social rejection" OR "social distance" OR "social isolation" OR "social marginali*" OR scapegoat* OR "structural violence" OR "differential treatment" OR embarrass* OR "social rejection[MeSH Terms]" OR "social stigma[MeSH Terms]" OR "prejudice[MeSH Terms]" OR "social distance[MeSH Terms]" OR "social isolation[MeSH Terms]" OR discrimination[MeSH Terms] "social discrimination[MeSH Terms]" OR "behavior, stereotyped[MeSH Terms]"

***Social contact*** *search terms*

"social contact" or "contact-based" or "direct contact" or "indirect contact" or testimon* or "personal contact" or "contact hypothesis" or "contact strateg*" or "contact theor*" or "intergroup contact" or "inter-group contact" or "personal interaction*" or "contact intervention*" or "interpersonal interaction*" OR "imagin* contact" OR "social media" OR video-based OR "mass media" OR storytel* OR "vicario* contact" OR "extended contact" OR film-based OR theatre* OR "expert testimonies[MeSH Terms]" OR "personal narratives[MeSH Terms]" OR "mass media[MeSH Terms]" OR multimedia[MeSH Terms] OR "social media[MeSH Terms]"

***Intervention*** *search terms*

Intervention* OR project* OR program* OR treatment OR promotion OR campaign* OR evaluation* OR activit* OR trial* OR "social marketing" OR therap* OR training* OR outreach OR monitor OR reduction OR approach* OR strateg* OR experiment* OR awareness OR pre-post OR initiativ* OR workshop OR service OR training OR "health education" OR "intervention studies[MeSH Terms]" OR "program evaluation[MeSH Terms]" OR "awareness[MeSH Terms]" OR "randomized controlled trials as topic[MeSH Terms]"

***Setting*** *search terms*

"low- and middle-income countr*" OR "low and middle-income countr*" OR "low income" OR "low-income" OR "middle income" OR "middle-income" OR LMIC* OR LAMIC OR "developing countr*" OR "third world" OR humanitarian OR "armed conflict" OR conflict* OR war* OR Afghanistan OR Albania OR Algeria OR "Antigua and Barbuda" OR "American Samoa" OR Angola OR Argentina OR Armenia OR Azerbaijan OR Bangladesh OR Barbados OR Belarus OR Belize OR Benin OR Bhutan OR Bolivia OR "Bosnia and Herzegovina" OR Botswana OR Brazil OR Bulgaria OR Burkina Faso OR Burundi OR "Cabo Verde" OR Cambodia OR Cameroon OR "Central African Republic" OR Chad OR Chile OR China OR Colombia OR Comoros OR Congo OR "Costa Rica" OR "Côte d'Ivoire" OR Cuba OR Cyprus OR Djibouti OR Dominica OR "Dominican Republic" OR Ecuador OR Egypt OR "El Salvador" OR "Equatorial Guinea" OR Eritrea OR Eswatini OR Ethiopia OR Fiji OR Gabon OR Gambia OR Georgia OR Ghana OR Gibraltar OR Greece OR Grenada OR Guatemala OR Guinea OR Guinea-Bissau OR Guyana OR Haiti OR Honduras OR Hungary OR India OR Indonesia OR Iran OR Iraq OR Jamaica OR Jordan OR Kazakhstan OR Kenya OR Kiribati OR Korea OR Kosovo OR "Kyrgyz Republic" OR Lao OR Lebanon OR Lesotho OR Liberia OR Libya OR "Macao SAR, China" OR Madagascar OR Malawi OR Malaysia OR Maldives OR Mali OR Malta OR "Marshall Islands" OR Mauritania OR Mauritius OR Mexico OR Micronesia OR Moldova OR Mongolia OR Montenegro OR Morocco OR Mozambique OR Myanmar OR Namibia OR Nepal OR New Caledonia OR Nicaragua OR Niger OR Nigeria OR "North Macedonia" OR Oman OR Pakistan OR Panama OR Papua OR "New Guinea" OR Paraguay OR Peru OR Philippines OR Poland OR Portugal OR "Puerto Rico" OR Romania OR "Russian Federation" OR Rwanda OR Samoa OR "São Tomé and Principe" OR Senegal OR Serbia OR Seychelles OR "Sierra Leone" OR "Solomon Islands" OR Somalia OR "South Africa" OR "South Sudan" OR "Sri Lanka" OR "St. Kitts and Nevis" OR "St. Lucia" OR "St. Vincent and the Grenadines" OR Sudan OR Surinam* OR "Syrian Arab Republic" OR Tajikistan OR Tanzania OR Thailand OR Timor-Leste OR Togo OR Tonga OR "Trinidad and Tobago" OR Tunisia OR Turkey OR Turkmenistan OR Tuvalu OR Uganda OR Uruguay OR Ukraine OR Uzbekistan OR Vanuatu OR Venezuela OR Vietnam OR "West Bank and Gaza" OR Yemen OR Zambia OR Zimbabwe OR "Ivory Coast" OR Russia OR Palestine OR "Palestinian Territories" OR "Bangla Desh" OR "West Bank" OR Gaza OR Syria

*Inclusion/exclusion criteria*

For the title/abstract screening phase and the full text screening phase, the following inclusion criteria were applied:

- Peer-reviewed publication
- Written in English/Dutch/French/Spanish/German
- Study is situated in a LMIC according to the World Bank classification list
- Describe an intervention in which social contact is used as a strategy for stigma reduction, regardless of what the stigmatized characteristics are
- Stigma (reduction) is measured quantitatively and/or qualitatively
- Participants are humans

The following exclusion criteria were applied:

- Published as book chapters, dissertations, reviews, grey literature, background articles
- Ineligible languages (everything which is not English/Dutch/French/Spanish/German)
- Study is situated in a HIC according to the World Bank classification list
- Social contact intervention is not mentioned in relation to stigma reduction
- Social contact was measured without deploying a social contact strategy (e.g. survey studies)
- Stigma (reduction) is not measured
- There is a social contact strategy, but it is unclear what the social contact exposure is (e.g. mass media exposure without knowing to what media people are exposed)
- An indirect intervention linking to social contact (such as social media exposure) is not deployed to bring people with and without stigmatizing characteristics into contact and aiming to getting to know each other and the stigmatizing characteristic
- Stigma occurs in two ways (e.g. two different religion groups), where there is no power imbalance
- Participants are not humans

Full text is not available after contacting the first author twice.
