# Supplementary material for: Social contact as a strategy to reduce stigma in low- and middle-income countries: A systematic review and expert perspectives
Source: PLOS Glob Public Health. 2024 Mar 27;4(3):e0003053. doi: 10.1371/journal.pgph.0003053 (PMC10971769; doi:10.1371/journal.pgph.0003053)
Supplement: S2 Text — (DOCX) [file pgph.0003053.s004.docx]

**Supplementary material S2 Text: qualitative topic guide**

**Research question:**What influences the choice of a social contact type?
What are facilitators and barriers of using social contact?
How was the interaction between people with and without the stigma supported?
What are lessons learnt?

*Start*

- Introduction interviewer
- Introduction project (social contact, stigma reduction, LMIC) + goal, create practical tool
- Beyond your manuscript(s)
- Recording and informed consent

*Background and (work) experience*

- What is your background and experience with social contact?

*Choice for types of social contact strategies*

- Why did you use social contact?
- What types of SC have you applied? And why?
  - (E.g. imagined/direct/indirect]
- Are there circumstances that influence the choice of social contact?
  - (E.g. context/culture, resources)

*General: using social contact*

- In your experience with social contact, what were barriers and facilitators to implement/start social contact as a strategy? Why?
- How did you mitigate the barriers?
- Did you encounter any unintended/harmful consequences due to SC? If so, what where these? Why did it happen?
- In how far have PWLE been part of shaping social contact?
- (Are there differences between types of social contact, and if so, what?)

*Supporting interaction during social contact*

- Is something done to create the circumstances in such a way that it optimizes the social contact? If so, what (before and during)?
- Did you do anything to support the interaction between people with and without the stigma? What did you do? Why?
  - If no, why not?
  - (E.g. comfortable rooms, rules of engagement, …)
- How made you sure that the interaction was impactful and harmless?

*Lessons learnt*

- What are your lessons learnt about using social contact strategies aiming to reduce stigma?
- In hindsight, what would you do differently next time?
- What advices do you have to fellow researchers or stigma-reduction-practitioners using social contact?
  - With in mind the practical tool we aim to create, what are do’s and don’ts regarding SC which should be on that tool?

*End*

- Any other things which have not been mentioned yet?
- Thank you for your participation
