# Supplementary material for: Social contact as a strategy to reduce stigma in low- and middle-income countries: A systematic review and expert perspectives
Source: PLOS Glob Public Health. 2024 Mar 27;4(3):e0003053. doi: 10.1371/journal.pgph.0003053 (PMC10971769; doi:10.1371/journal.pgph.0003053)
Supplement: S3 Text — (DOCX) [file pgph.0003053.s005.docx]

**Supplementary material S2 Table: Details of quality appraisal according to JBI**

| **Quasi-experimental JBI checklist** | | | **Q1** | | | | | **Q2²** | | **Q3²** | | | **Q4²** | | | **Q5** | | | | **Q6** | | | **Q7** | | | **Q8**¹ | | | | | **Q9** | | | **Overall appraisal** | |
| --- | --- | --- | --- | --- | --- | --- | --- | --- | --- | --- | --- | --- | --- | --- | --- | --- | --- | --- | --- | --- | --- | --- | --- | --- | --- | --- | --- | --- | --- | --- | --- | --- | --- | --- | --- |
| Ahuja 2017 | | | Yes | | | | | Yes | | N/A | | | N/A | | | No | | | | No | | | Yes | | | Unclear | | | | | Unclear | | | Moderate | |
| Altindag 2006 | | | Yes | | | | | Yes | | Yes | | | Yes | | | Yes | | | | Unclear | | | Yes | | | Yes | | | | | Unclear | | | Moderate | |
| Apindecha 2007 | | | Yes | | | | | Yes | | Yes | | | Yes | | | No | | | | Unclear | | | Yes | | | Unclear | | | | | Unclear | | | Moderate | |
| Bagci 2020 | | | Yes | | | | | Yes | | Yes | | | Yes | | | No | | | | Unclear | | | Yes | | | Unclear | | | | | Yes | | | Moderate | |
| *Chidrawi 2014(/Chidrawi 2016/French 2014/French 2015)³* | | | *Yes* | | | | | *Yes* | | *N/A* | | | *N/A* | | | *Yes* | | | | *Unclear* | | | *Yes* | | | *Unclear* | | | | | *Unclear* | | | *Moderate* | |
| Chidrawi 2016(/Chidrawi 2014/French 2014/French 2015)³ | | | Yes | | | | | Yes | | N/A | | | N/A | | | Yes | | | | Unclear | | | Yes | | | Unclear | | | | | Unclear | | | Moderate | |
| Duman 2017 | | | Yes | | | | | Yes | | Yes | | | Yes | | | No | | | | Unclear | | | Yes | | | Unclear | | | | | Unclear | | | Moderate | |
| De Groot 2021a | | | Yes | | | | | Yes | | Yes | | | No | | | No | | | | Unclear | | | Yes | | | Unclear | | | | | Yes | | | Moderate | |
| De Groot 2021b | | | Yes | | | | | Yes | | Yes | | | No | | | No | | | | Unclear | | | Yes | | | Yes | | | | | Yes | | | Moderate | |
| Hofmann-Broussard 2007 | | | Yes | | | | | Yes | | Yes | | | Yes | | | No | | | | Unclear | | | Yes | | | Unclear | | | | | Yes | | | Moderate | |
| Jain 2013 | | | Yes | | | | | Yes | | N/A | | | N/A | | | No | | | | Yes | | | Yes | | | Yes | | | | | Unclear | | | Moderate | |
| Kohrt 2020(/Rai 2018)³ | | | Yes | | | | | Yes | | N/A | | | N/A | | | Yes | | | | Yes | | | Yes | | | Unclear | | | | | Unclear | | | Moderate | |
| Logie 2021(/Logie 2022) | | | Yes | | | | | Yes | | N/A | | | N/A | | | No | | | | N/A | | | Yes | | | Unclear | | | | | Yes | | | Moderate | |
| *Logie 2022(/Logie 2021)* | | | *Yes* | | | | | *Yes* | | *N/A* | | | *N/A* | | | *Yes* | | | | *Yes* | | | *Yes* | | | *Unclear* | | | | | *Yes* | | | *Moderate* | |
| Maulik 2019(/Maulik 2017)³ | | | Yes | | | | | Yes | | N/A | | | N/A | | | Yes | | | | Yes | | | Yes | | | Unclear | | | | | Yes | | | High | |
| *Maulik 2017(/Maulik 2019)³** | | | *Yes* | | | | | *Yes* | | *N/A* | | | *N/A* | | | *No* | | | | *Yes* | | | *Yes* | | | *Unclear* | | | | | *Unclear* | | | *Moderate* | |
| Ng 2017 | | | Yes | | | | | Yes | | N/A | | | N/A | | | No | | | | Yes | | | Yes | | | Unclear | | | | | Yes | | | Moderate | |
| Nistor 2021 | | | Yes | | | | | Unclear | | Unclear | | | Yes | | | No | | | | Unclear | | | No | | | Unclear | | | | | Unclear | | | Low | |
| Pufahl 2021 | | | Yes | | | | | Yes | | N/A | | | N/A | | | No | | | | No | | | Yes | | | Unclear | | | | | Unclear | | | Moderate | |
| *Rimal 2008(/Creel 2011)³* | | | *Yes* | | | | | *Yes* | | *N/A* | | | *N/A* | | | *No* | | | | *Unclear* | | | *Yes* | | | *Yes* | | | | | *Yes* | | | *Moderate* | |
| Rong 2011 | | | Yes | | | | | Yes | | Yes | | | Yes | | | Yes | | | | Unclear | | | Yes | | | Unclear | | | | | Unclear | | | Moderate | |
| Sakalli 2003 | | | Yes | | | | | Yes | | Yes | | | Yes | | | No | | | | Unclear | | | Yes | | | Unclear | | | | | Unclear | | | Moderate | |
| Schloegel 2016 | | | Yes | | | | | Yes | | Yes | | | Yes | | | No | | | | Yes | | | Yes | | | Unclear | | | | | Unclear | | | Moderate | |
| Shah 2014 | | | Yes | | | | | Yes | | Yes | | | Yes | | | No | | | | Yes | | | Yes | | | Unclear | | | | | Unclear | | | Moderate | |
| Shah 2015 | | | Yes | | | | | Yes | | N/A | | | N/A | | | No | | | | Yes | | | Yes | | | Unclear | | | | | Unclear | | | Moderate | |
| Tercan 2021* | | | Yes | | | | | Yes | | Yes | | | Yes | | | No | | | | Yes | | | Yes | | | Unclear | | | | | Unclear | | | Moderate | |
| Uys 2009* | | | Yes | | | | | Yes | | N/A | | | N/A | | | No | | | | Yes | | | Yes | | | Unclear | | | | | Unclear | | | Moderate | |
| West 2015 | | | Yes | | | | | Yes | | Yes | | | Yes | | | No | | | | Unclear | | | Yes | | | Unclear | | | | | Unclear | | | Moderate | |
| Wu 2008 | | | Yes | | | | | Yes | | No | | | Yes | | | Yes | | | | No | | | Yes | | | Unclear | | | | | Unclear | | | Moderate | |
| **RCT JBI checklist** | | **Q1** | | | **Q2** | **Q3** | | | **Q4** | | | **Q5** | | | **Q6** | | **Q7¹** | | **Q8** | | | **Q9** | | | **Q10** | | **Q11**¹ | | **Q12** | | | **Q13** | | | **Overall appraisal** |
| Ahuja 2019 | | Yes | | | Unclear | Yes | | | Unclear | | | Unclear | | | Unclear | | Yes | | No | | | Yes | | | Yes | | Unclear | | Unclear | | | Yes | | | Moderate |
| Arthur 2020a(/Arthur 2020b)³ | | Yes | | | Yes | Yes | | | Unclear | | | Unclear | | | Unclear | | Yes | | Yes | | | Yes | | | Yes | | Unclear | | Yes | | | Yes | | | Moderate |
| *Arthur 2020b(/Arthur 2020a)³* | | *Yes* | | | *Yes* | *Yes* | | | *Unclear* | | | *Unclear* | | | *Unclear* | | *Yes* | | *Yes* | | | *Yes* | | | *Yes* | | *Unclear* | | *Yes* | | | *Yes* | | | *Moderate* |
| Banerjee 2015 | | Yes | | | Unclear | Yes | | | Unclear | | | Unclear | | | Unclear | | Yes | | Yes | | | Unclear | | | Yes | | Yes | | No | | | Yes | | | Moderate |
| Carvalho-Freitas 2017 | | Unclear | | | Unclear | Unclear | | | Unclear | | | Unclear | | | Unclear | | Yes | | N/A | | | Yes | | | Yes | | Unclear | | Unclear | | | Yes | | | Low |
| Creel 2011(/Rimal 2008)³ | | Unclear | | | Unclear | Yes | | | Unclear | | | Unclear | | | Unclear | | Yes | | Yes | | | Unclear | | | Yes | | Unclear | | Yes | | | Yes | | | Moderate |
| Dadun 2017(/Peters 2015/Peters 2016)³ | | Yes | | | Unclear | Yes | | | Unclear | | | Unclear | | | Unclear | | Yes | | Yes | | | Unclear | | | Yes | | Unclear | | Yes | | | Yes | | | Moderate |
| Fernandez 2016 | | Yes | | | Yes | Yes | | | Unclear | | | Unclear | | | Unclear | | Yes | | Yes | | | Yes | | | Yes | | Unclear | | Unclear | | | Yes | | | Moderate |
| Finkelstein 2008 | | Yes | | | Unclear | Yes | | | Unclear | | | Unclear | | | Unclear | | Yes | | No | | | Yes | | | Yes | | Unclear | | Yes | | | Yes | | | Moderate |
| Gürbüz 2020 | | Yes | | | Unclear | Yes | | | Unclear | | | Unclear | | | Unclear | | Yes | | No | | | Yes | | | Yes | | Unclear | | Unclear | | | Yes | | | Moderate |
| Kohrt 2021 | | Yes | | | Yes | Unclear | | | Yes | | | Unclear | | | Yes | | Unclear | | Yes | | | Yes | | | Yes | | Unclear | | Yes | | | Yes | | | Moderate |
| Ozaydin 2021 | | Yes | | | Yes | Yes | | | Unclear | | | Unclear | | | Yes | | Yes | | Yes | | | Yes | | | Yes | | Unclear | | Yes | | | Yes | | | Moderate |
| Pekçetin 2021 | | Yes | | | Yes | Yes | | | Unclear | | | Unclear | | | Yes | | Yes | | Yes | | | Unclear | | | Yes | | Unclear | | Yes | | | Yes | | | Moderate |
| *Peters 2015(/Dadun 2017/Peters 2016)³* | | *Unclear* | | | *Unclear* | *Unclear* | | | *Unclear* | | | *Unclear* | | | *Unclear* | | *Yes* | | *Yes* | | | *Unclear* | | | *Yes* | | *Unclear* | | *Yes* | | | *Yes* | | | *Moderate* |
| Ran 2022 | | Yes | | | Yes | Yes | | | Yes | | | No | | | Yes | | Yes | | Yes | | | Yes | | | Yes | | Unclear | | Yes | | | Yes | | | High |
| Tergesen 2021* | | Yes | | | Yes | Unclear | | | Unclear | | | Unclear | | | Unclear | | Yes | | Yes | | | Yes | | | Yes | | Unclear | | Yes | | | Yes | | | Moderate |
| Vaghee 2018 | | Unclear | | | Unclear | Yes | | | Unclear | | | Unclear | | | Unclear | | Yes | | Unclear | | | Unclear | | | Yes | | Unclear | | Yes | | | Yes | | | Moderate |
| Zhang 2022 | | Yes | | | Yes | Yes | | | Unclear | | | Unclear | | | Unclear | | Yes | | Yes | | | Yes | | | Yes | | Unclear | | Yes | | | Yes | | | Moderate |
| **Qualitative study JBI checklist** | **Q1** | | | **Q2** | | | **Q3** | | | | **Q4** | | | **Q5** | | | | **Q6** | | | **Q7** | | | **Q8** | | | | **Q9** | | **Q10** | | | **Overall appraisal** | | |
| Doostri-Irani 2017 | Yes | | | No | | | No | | | | No | | | Yes | | | | No | | | No | | | No | | | | No | | Yes | | | Low | | |
| *French 2014(/Chidrawi 2014/ Chidrawi 2016/French 2015)³* | *Yes* | | | *Yes* | | | *Yes* | | | | *Yes* | | | *Yes* | | | | *No* | | | *No* | | | *Yes* | | | | *Yes* | | *Unclear* | | | *Moderate* | | |
| *French 2015(/Chidrawi 2014/ Chidrawi 2016/French 2014)³* | *Yes* | | | *Yes* | | | *Yes* | | | | *Yes* | | | *Yes* | | | | *No* | | | *No* | | | *Yes* | | | | *Yes* | | *Unclear* | | | *Moderate* | | |
| *Kaiser 2022(/Kohrt 2021)* | *Yes* | | | *Yes* | | | *Yes* | | | | *Yes* | | | *Yes* | | | | *No* | | | *No* | | | *Yes* | | | | *Yes* | | *Yes* | | | *High* | | |
| Logie 2019 | Yes | | | Yes | | | Yes | | | | Yes | | | Yes | | | | No | | | No | | | Yes | | | | Yes | | Yes | | | Moderate | | |
| *Maulik 2017(/Maulik 2019)³** | *Yes* | | | *Yes* | | | *Yes* | | | | *Yes* | | | *Yes* | | | | *No* | | | *No* | | | *Yes* | | | | *Yes* | | *Yes* | | | *Moderate* | | |
| *Peters 2016(/Dadun 2017/Peters 2015)³* | *Yes* | | | *Yes* | | | *Yes* | | | | *Yes* | | | *Yes* | | | | *Yes* | | | *Yes* | | | *No* | | | | *Yes* | | *Yes* | | | *High* | | |
| Prinsloo 2016 | Yes | | | Unclear | | | Yes | | | | Yes | | | Yes | | | | No | | | No | | | Yes | | | | Yes | | Yes | | | Moderate | | |
| *Rai 2018(/Kohrt 2020)³* | *Yes* | | | *Yes* | | | *Yes* | | | | *Yes* | | | *Yes* | | | | *No* | | | *No* | | | *Yes* | | | | *Yes* | | *Yes* | | | *Moderate* | | |
| Tercan 2021* | Yes | | | Yes | | | Yes | | | | Yes | | | Yes | | | | No | | | No | | | Yes | | | | Unclear | | Yes | | | Moderate | | |
| Tergesen 2021* | Yes | | | Yes | | | Yes | | | | Yes | | | Yes | | | | No | | | No | | | Yes | | | | Yes | | Yes | | | Moderate | | |
| Uys 2009* | No | | | No | | | Unclear | | | | No | | | No | | | | No | | | No | | | No | | | | Yes | | No | | | Low | | |

**Legend***High* quality was considered when more than 85% of the total of relevant questions were answered with a “yes”, *moderate* quality when 40-85% of the total of relevant questions were answered with a “yes”, and *low* quality when less than 40% of the total of relevant questions were answered with a “yes”. Relevant questions are defined as questions which can be answered due to the nature of the study (i.e., some questions are not answerable for one-arm-studies², and therefore considered as irrelevant). When one study has more publications³, all publications are assessed: the main publication is in black, and the other side publications are in grey.

* Stigma was measured quantitatively as well as qualitatively. Therefore, when a study used mixed methods, it is assessed with a quantitative and a qualitative checklist. When there were qualitative methods conducted for other reasons than investigating stigma (such as examining general opinions about the intervention), the study was not further assessed with a JBI qualitative checklist.
¹ Self-reported questionnaires were understood as follows: if it was made sure that participants understood the questions well, this was judged as a “yes”. Otherwise, it was judged as “unclear” (in case of limitely or not reported) or “no” (in the rare case if it was reported that this was not done).
