# Supplementary material for: Social contact as a strategy to reduce stigma in low- and middle-income countries: A systematic review and expert perspectives
Source: PLOS Glob Public Health. 2024 Mar 27;4(3):e0003053. doi: 10.1371/journal.pgph.0003053 (PMC10971769; doi:10.1371/journal.pgph.0003053)
Supplement: Attachment — Submitted filename: Response to Reviewers_Manuscript SC_PLOSGPH_R1 (Oct2023).docx [file pgph.0003053.s006.docx]

RESPONSE TO REVIEWERS

5. Review Comments to the Author

Please use the space provided to explain your answers to the questions above. You may also include additional comments for the author, including concerns about dual publication, research ethics, or publication ethics. (Please upload your review as an attachment if it exceeds 20,000 characters)

**REVIEWER 1**

Reviewer #1: This reviewer thought providing a high-level review focusing on broader conceptual and methodological issues might be helpful. I have stayed away from providing feedback section by section because it may be redundant if the major/broader issues in this manuscript are addressed.

Since stigma is linked to, among other factors, discrimination and social isolation of specific individuals, exploring the role of social contact in preventing or reducing the adverse effects of stigma on health outcomes is an essential contribution with significant theoretical, empirical, and practical implications. I commend the authors for engaging in such a significant and productive research study.

However, outlined below are some of the major conceptual and methodological issues that need to be addressed.

1. There is a need for clarity and perhaps consistency on the current study’s research question and broad objective/goal. In the abstract, the authors state, “Social contact (SC) has been identified as a promising strategy for stigma reduction.” The following study aim in line 32 of the abstract section says the current study is a systematic review that assesses the effectiveness of social contact (SC) as a stigma reduction strategy. However, the study goals in the introduction (see lines 111 to 116 of the introduction section) are inconsistent with the above-stated study aim. Instead, they read like objectives of a scoping review (I address this last aspect more in the next point.

**[RESPONSE TO REVIEWER]**

Thank you. This review builds upon previous, narrower (one stigma) systematic reviews. The protocol for this review was registered within and accepted by PROSPERO, the database to register and check the methodological criteria of proposed systematic reviews and check the methodological criteria for a systematic review. This review has clearly delineated inclusion (and exclusion) criteria, with “stigma reduction interventions including a social contact component in LMIC” to be included. We took the opportunity to add some explorative work on the topic.

To clarify this in the introduction, we have rewritten this section to make clear that the main study is a systematic review which has been complemented with expert perspectives. Please see the abstract (line 38) and the introduction (lines 102-112).

2. Based on study objectives (see lines 111 to 116 of the introduction section) and the lack of specificity in outcomes of interests, this review should be a scoping review. For instance, no objective/question explicitly talks about the effectiveness of social contact in stigma reduction. As we say in research, the question drives the method. I also suggest that the authors review the research questions and methodologies used for different review types to make informed choices about whether to conduct a scoping review or revise the research questions to align with the research method—a systematic review.

**[RESPONSE TO REVIEWER]**

The main and initial question driving this review was to create an overview of all LMIC-based stigma reduction interventions that apply a social contact strategy, to assess both content and effectiveness. This was described in lines 111-112 (original manuscript). But to highlight this better, we made clarifying edits in the manuscript to differentiate between the main aim and the supportive questions. See our response to Comment 1.

3. Suppose the authors are interested in conducting a systematic review. In that case, they need to specify the aspect of stigma regarding the related health condition (i.e., mental health stigma, HIV/AIDS stigma, sexual violence). Choosing a specific type of stigma concerning a health condition allows the authors to make their research objectives specific enough to be measurable and testable. Although 41 studies found SC effective in reducing stigma, it is unclear how this effect is distributed across different health conditions (i.e., sexual violence, mental health, or HIV/AIDS).

**[RESPONSE TO REVIEWER]**

Thank you. We approached stigma as the main issue, irrespective of it being linked to having experienced sexual violence, a mental health condition, HIV/AIDS etc., as outlined in our PROSPERO protocol (CRD42022311676). We did so as we were guided by recent research^[1]^ that encourages researchers and practitioners to step away from a siloed approach towards stigma (focusing on one stigma (category)), as, besides the differences, across stigmas there are many overlaps in stigma dynamics, manifestations and strategies, for example. We wanted to learn more about the application of social contact to reduce stigmatisation and how it has been applied across stigmas, so that the different ‘silos’ can build on each other's knowledge. By adding a systematic review across stigmas, we are contributing to the knowledge base which is up to this moment quite siloed still (e.g. focusing only on mental health stigma in literature reviews).

Additionally, indeed, most of the studies (38 studies, not 41 studies, as mentioned in line 375) have shown effectiveness in reducing stigma, however, as our systematic review has demonstrated, not all these studies applied rigour to determine effectiveness. Furthermore, while figure 5 highlighted all studies and their level of significance and showed that 8 studies out of 44 are statistically significant, we realise that we did not go in-depth on these statistically significant studies.

To ensure that this finding from the review is clearer, we have made clarifying edits and added some information in the results and discussion section:

- Results: lines 373-390; lines 397 – 404; and lines 414 – 416
- Discussion lines 552-563

4. Additionally, it enables the authors to discuss specific adverse impacts of stigma on a health condition and ways in which social contact (as opposed to, maybe, social isolation?) could mitigate those adverse effects or promote positive health outcomes in that particular condition. As of now, even the introduction section needs to be narrower.

**[RESPONSE TO REVIEWER]**

Thank you for the suggestion to go more in-depth of the adverse impacts of stigma on a health condition. As this goes beyond the focus and realm of our current review and would enlarge the paper drastically, we have opted not to do this.

However, we see the value of your comment and have integrated this into the limitations of this review. Please see lines 616-618.

5. In any review, it is important to discuss existing knowledge and gaps in existing reviews in the introduction to establish the relevance of the current study. I recommend that the authors of this systematic review consider adding that aspect to the introduction section in a paragraph that precedes the one on the study goal and research objectives.

**[RESPONSE TO REVIEWER]**

Thank you. Thanks to your point we have reformulated this section in the introduction to showcase the gaps more clearly. Please see lines 87-100.

6. Although this could be refreshingly innovative, it is still being determined why the authors used a mixed methods study that combines a systematic review with a qualitative inquiry. The rationale of this method needs to be explained in the introduction. Nor is it reflected in the research objectives in lines 111 to 116 of the introduction section. In other words, provide clarity on which research questions are addressed by the systematic review and which one will be qualitatively assessed. Additionally, when one uses a mixed methods study, it is essential to specify in the methods section which part of the mix is the major, which is the minor, and how they relate to each other.

**[RESPONSE TO REVIEWER]**

Thank you. The systematic review is the major data collection method, which we complemented with interviews to get more context and in-depth information from researchers/authors on their perspectives around the use of social contact as this is not often described in detail in the papers, while this can be of importance in the implementation of social contact, either in being effective or (not) harmful. In relation to Comment 1, we have now clarified in the introduction that the main question is answered by the literature, while sub-questions a and b were answered by information retrieved from both the review and the interviews. See lines 104-112. Additionally, we have adjusted the phrasing in the abstract (methods), in the Methods section (part 2) (lines 187-189), Discussion (line 518; line 602) .

7. Authors need an operational definition of social contact.

**[RESPONSE TO REVIEWER]**

Thank you for highlighting we did not clarify this sufficiently. Please see lines 74-76 for an adjustment.

**REVIEWER 2**

Reviewer #2: The study exhibited a well-conceived and meticulously executed research design. The team's endeavor to investigate the utilization of social contact strategies within low- and middle-income countries (LMICs) was both innovative and commendable, effectively addressing a critical knowledge gap for public health stakeholders.

The incorporation of a systematic review alongside interviews with the authors further bolstered the study's robustness and depth. This dual methodology approach contributed to the overall strength of the research.

Certainly, here are your suggestions rephrased in a professional manner for an academic review:

1. Regarding the Main Table: Consider reorganizing the study data in a chronological and, if feasible, regional order within the main table. This adjustment would facilitate a clearer understanding for readers of the geographical focus throughout the article.

**[RESPONSE TO REVIEWER]**

Thank you. The focus of this systematic review was to understand more about the implementation of social contact. The main table has been organised per social contact type, which we would like to keep. As the regional perspective is secondary, we opt not to organise per region.

However, we saw your point of additional ordering within the main table, and have therefore ordered, underneath each social contact category, the studies per stigma category (mental health; physical health; not health-related; and various) and then alphabetically). Please see table 1 in the Results Section.

2. Concerning the Results Section: Ensure consistency in the reporting of frequencies and percentages. Some instances display variations such as (n=0, 0%), 0(0%), or solely reporting 'n' or percentage values. Uniformity in this aspect would enhance the clarity of the findings.

**[RESPONSE TO REVIEWER]**

Thank you – based on your feedback we decided to use two types of write up: (n=x; x%) and number (x%), this also to help sometimes the understanding of which total number was represented. We have adjusted some sections where we had written % (number) (see lines 4073-416).

3. Is there a specific reason why the authors did not include schizophrenia, depression, autism, and OCD within the category of mental health conditions in the study?

**[RESPONSE TO REVIEWER]**

Thank you for highlighting this, this was an oversight, specifically in Table 1, as these conditions were indeed included although not always overtly mentioned. We have ensured that all mental health conditions are categorised underneath mental health.

4. Regarding the Author's Inclusion of Literature in the Results Section: Clarify the rationale behind the inclusion of literature references within the results section (lines 349 to 355). This could be elaborated upon to justify its presence within this section.

**[RESPONSE TO REVIEWER]**

Thank you. We have taken out the literature references in the written sections of the results.

However, we have kept the references in Table 2, as it provides an overview of all positive factors and their corresponding frameworks. We want to keep these references in to do justice to their origin.

5. Inconsistencies in Reference Style: Address inconsistencies in reference style observed in the manuscript (lines 349, 521, 526, 527, 532, 533, 535, 536, 550, and 590). A uniform reference style should be implemented throughout the document for consistency and adherence to academic conventions.

**[RESPONSE TO REVIEWER]**

Thank you for spotting this. We have checked and adjusted these.

**REVIEWER 3**

Reviewer #3: Great work by the authors team, writeup show academic fashion in research. Some minor queries.

1. Multiple places its mentioned that 10% of the records were independently reviewed by both authors (Please crosscheck)

**[RESPONSE TO REVIEWER]**

Thank you for your review. Thanks to your comment, we realised that we actually did more independent reviewing of records, data extraction and analysis checks and made the necessary adjustments. The %s described basically came from our PROSPERO protocol.

2.How did research team managed to review articles from 5 languages

**[RESPONSE TO REVIEWER]**

The two main authors share these languages among each other. Additionally, only English-language articles were eligible for inclusion.

3. This study can be splitted as two independent study, is there any specific reason to write such a huge paper.

**[RESPONSE TO REVIEWER]**

Thank you. The explorative interviews conducted were done to solicit the perspective of the author experts to retrieve more in-depth information complementary to was reported upon within the included studies. The interviews were really an add-on to the systematic review and therefore we have chosen to keep them together.

4. In title (explorative qualitative study) is little confusing for the readers hence kindly think about it. (Social contact as a strategy to reduce stigma in low- and middle-income countries: a systematic review and exploration on author's perspective)

**[RESPONSE TO REVIEWER]**

Thank you for this suggestion, we now propose the title: Social contact as a strategy to reduce stigma in low- and middle-income countries: a systematic review and expert perspectives.

Additionally to this point, we have made some references throughout the manuscript on soliciting input from experts.

6. PLOS authors have the option to publish the peer review history of their article ([what does this mean?](https://journals.plos.org/globalpublichealth/s/editorial-and-peer-review-process#loc-peer-review-history)). If published, this will include your full peer review and any attached files.

**Do you want your identity to be public for this peer review?** If you choose “no”, your identity will remain anonymous but your review may still be made public.

For information about this choice, including consent withdrawal, please see our [Privacy Policy](https://www.plos.org/privacy-policy).

Reviewer #1: **Yes:** THABANI NYONI

Reviewer #2: No

Reviewer #3: No

1. 1) Van Brakel, W. H., Cataldo, J., Grover, S., Kohrt, B. A., Nyblade, L., Stockton, M., ... & Yang, L. H. (2019). Out of the silos: identifying cross-cutting features of health-related stigma to advance measurement and intervention. *BMC medicine*, *17*, 1-17; 2) Stangl, A. L., Earnshaw, V. A., Logie, C. H., Van Brakel, W., C. Simbayi, L., Barré, I., & Dovidio, J. F. (2019). The Health Stigma and Discrimination Framework: a global, crosscutting framework to inform research, intervention development, and policy on health-related stigmas. *BMC medicine*, *17*, 1-13
