# Supplementary material for: Social contact as a strategy to reduce stigma in low- and middle-income countries: A systematic review and expert perspectives
Source: PLOS Glob Public Health. 2024 Mar 27;4(3):e0003053. doi: 10.1371/journal.pgph.0003053 (PMC10971769; doi:10.1371/journal.pgph.0003053)
Supplement: Attachment — Submitted filename: Response to Reviewers R2 (Feb2024).docx [file pgph.0003053.s007.docx]

Response to Reviewers – Resubmission 1 Social Contact Systematic Review

(February 2024)

**Comment 1**

I have carefully examined the revised manuscript. The study has been restructured with a clear focus on its aims and objectives. However, as highlighted by the first reviewer, it would have been beneficial to limit the scope of the study to a single condition—either Mental Health, LGBTQ, Albinism, or HIV—in order to more accurately assess the effectiveness of the Social Contact Strategy. Your analysis convincingly underscores the importance of reporting validity to ascertain the strategy's effectiveness, yet it is important to acknowledge that this effectiveness may vary depending on the condition being addressed. Therefore, it is advisable to incorporate this observation into both the discussion and the conclusion sections. I recommend that you include the finding—that the majority of the studies reviewed applied the Social Contact Strategy in the context of mental health—within the discussion (specifically, adding to line 550) and conclusion sections, as it could guide future research directions.

**Response**

Thank you for your comment. When we started this study, we followed recommendations by older (Heijnders & Van Der Meij, 2006) and recent research (Kemp et al., 2019; Stangl et al., 2019; van Brakel et al., 2019) to look at stigmas beyond siloes because there is a lot to learn across stigmas (mechanisms, consequences but also strategies). Therefore we thought it to be more beneficial to look at the application of social contact across stigmas than in one single condition. Additionally, multiple reviews have already focused on social contact and mental health.

Your comment indicates that social contact might be more effective for one stigma (e.g. Mental health) than another (e.g. LGTBQI, as a non-health related stigma or HIV as a physical health-related stigma) is very interesting. With that question in mind, we looked at the data again, zooming in on the stigma category and the social contact type.

1. First, we looked at all studies (single or multiple arm studies) from two perspectives: 1) the stigma category (mental health; physical health and non health-related stigmas) and 2) the contact type (e.g. direct contact; indirect contact etc). We examined how they reported their outcomes for main effect of time, main effect of group (multiple arms studies only) and interaction effect (multiple arm studies only) (significant, not significant or not reported). We compared the interventions to the same category (so mental health interventions with mental health interventions) and type (so direct contact with direct contact interventions).
   - For the stigma category that e.g. means:
     - Main effect of time: 84% of the mental health stigma interventions reported significance; meaning that 16% did not (not significant or no reporting).
     - Main effect of group: between 0% and 29% reported significance (and 71+% did not)
     - Interaction effect: 38% of the non health-related stigma intervention studies reported significant interaction effects against 29% of mental health stigma intervention studies.
   - For the social contact type that e.g. means:
   - Main effect of time: 64% of the indirect contact interventions reported significance (36% did not).
   - Main effect of group: between 0% and 40% of the SC reported significance (60+% did not).
   - Interaction effect: between 0% (collaborative; imagined/vicarious) and 33% (indirect contact reported significance (the majority did not report on this).

See tables and figures below for further information. We have included these figures as Figure 5a and Figure 5b (and removed the original Figure 5).

| **Significance reported (time; group; interaction) from the stigma category perspective** | **Significance reported (time; group; interaction) from the contact type perspective** |
| --- | --- |
|  |  |
|  |  |

2. Then, we looked specifically at the eight interventions reporting significant interaction effects, combining the social contact type and the stigma category. This demonstrates the following, e.g.:
   - Stigma category perspective:
     - 100% (n=1: direct contact) of the interventions addressing multiple stigmas reported significance, and 38% (n=3: all direct contact) of the non health-related stigma interventions (n=8: 5 direct, 1 imagined, 1 vicarious, 1 indirect) did
     - 28% (n=4: indirect contact (n=3), combination of SC (n=1) of the mental health-related stigma interventions (n=14: 6 indirect, 4 direct and 4 indirect) did
     - None of the physical health stigmas reported (significance)
   - Social contact type perspective:
     - 33% (n=4: 3 non health-related, 1 multiple stigmas) of the direct contact type interventions (n=12: 5 non health-related, 4 mental health, 2 physical health, 1 multiple) reported significant interaction effect
     - 30% (n=3; all mental health) of the indirect contact type interventions (n=10: 6 mental health, 2 physical health, 1 non health-related) did
     - 20% (n=1; mental health) of the combined contact type interventions (n=5; 4 mental health, 1 physical health)) did.

This shows that only 28% of mental health stigma reduction interventions applying any form of social contact showed statistical significant outcome through interaction effects.

This also shows that one-third or less of all contact intervention types demonstrated statistically significant outcomes through interaction effects, with direct contact leading with 33% followed by indirect.

See tables and figures below for further information. We have included these figures as Figure 6a and Figure 6b.

| **From Stigma Category perspective** | **From Social Contact Type perspective** |
| --- | --- |
|  |  |
|  |  |

Taking the above into account, we have made changes in:

1) The abstract (as we added some information, we also adjusted other parts of the abstract to remain within 300 words)
2) Results – Effectiveness of SC interventions paragraph (lines 392-433)
3) Discussion – lines 552-553; lines 5580 (the original 550)-596
4) Conclusions – lines 657-666

Heijnders, M., & Van Der Meij, S. (2006). The fight against stigma: an overview of stigma-reduction strategies and interventions. In *Psychology, health & medicine* (Vol. 11, Issue 3, pp. 353–363). https://doi.org/10.1080/13548500600595327

Kemp, C. G., Jarrett, B. A., Kwon, C. S., Song, L., Jetté, N., Sapag, J. C., Bass, J., Murray, L., Rao, D., & Baral, S. (2019). Implementation science and stigma reduction interventions in low- and middle-income countries: A systematic review. In *BMC Medicine* (Vol. 17, Issue 1). BioMed Central Ltd. https://doi.org/10.1186/s12916-018-1237-x

Stangl, A. L., Earnshaw, V. A., Logie, C. H., van Brakel, W., Simbayi, L. C., Barré, I., Dovidio, J. F., C. Simbayi, L., Barré, I., & Dovidio, J. F. (2019). The Health Stigma and Discrimination Framework: a global, crosscutting framework to inform research, intervention development, and policy on health-related stigmas. *BMC Medicine*, *17*(1), 31. https://doi.org/10.1186/s12916-019-1271-3

van Brakel, W., Cataldo, J., Grover, S., Kohrt, B. A., Nyblade, L., Stockton, M., Wouters, E., & Yang, L. H. (2019). Out of the silos: Identifying cross-cutting features of health-related stigma to advance measurement and intervention. *BMC Medicine*, *17*(1). https://doi.org/10.1186/s12916-018-1245-x
